# *In Vitro* Activity of Daptomycin against *Enterococcus faecalis* under Various Conditions of Growth-Phases, Inoculum and pH

**DOI:** 10.1371/journal.pone.0064218

**Published:** 2013-05-21

**Authors:** Xavier Argemi, Yves Hansmann, Daniel Christmann, Sophie Lefebvre, Benoit Jaulhac, François Jehl

**Affiliations:** 1 Infectious Diseases Department, Strasbourg University Hospital, Strasbourg, France; 2 Infectious Diseases Department, Strasbourg University Hospital, Strasbourg, France; 3 Infectious Diseases Department, Strasbourg University Hospital, Strasbourg, France; 4 Institute of Bacteriology, Faculty of Medicine, Louis Pasteur University and Strasbourg University Hospital, Strasbourg, France; 5 Institute of Bacteriology, Faculty of Medicine, Louis Pasteur University and Strasbourg University Hospital, Strasbourg, France; 6 Institute of Bacteriology, Faculty of Medicine, Louis Pasteur University and Strasbourg University Hospital, Strasbourg, France; Kansas State University, United States of America

## Abstract

*Enterococcus faecalis* (*E. faecalis*) has become a major leading cause of nosocomial endocarditis. Treatment of such infections remains problematic and new therapeutic options are needed. Nine *E. faecalis* strains were tested: six obtained from patients presenting endocarditis, one with isolated bacteremia, and two reference strains. Antibiotics included daptomycin, alone or in combination, linezolid, tigecycline, rifampicin, gentamicin, teicoplanin, ceftriaxone and amoxicillin. Time-kill studies included colony counts at 1, 4 and 24 h of incubation. Significant bactericidal activity was defined as a decrease of ≥3log_10_CFU/ml after 24 h of incubation. Antibiotics were tested at a low (10^6^ CFU/ml) and high (10^9^ CFU/ml) inoculum, against exponential- and stationary-phase bacteria. We also performed time kill studies of chemically growth arrested *E. faecalis*. Various pH conditions were used during the tests. In exponential growth phase and with a low inoculum, daptomycin alone at 60 µg/ml and the combination amoxicillin-gentamicin both achieved a 4-log_10_ reduction in one hour on all strains. In exponential growth phase with a high inoculum, daptomycin alone was bactericidal at a concentration of 120 µg/ml. All the combinations tested with this drug were indifferent. In stationary phase with a high inoculum daptomycin remained bactericidal but exhibited a pH dependent activity and slower kill rates. All combinations that did not include daptomycin were not bactericidal in conditions of high inoculum, whatever the growth phase. The results indicate that daptomycin is the only antibiotic that may be able of overcoming the effects of growth phase and high inoculum.

## Introduction

Invasive infections with *Enterococcus faecalis* (*E. faecalis*) are infrequent but steadily increasing, especially in elderly or immunocompromised patients, and in the field of health-care-associated infections [1]. The incidence of infective endocarditis is 3 to 10 per 100,000 people per year on average; 5–11% of these infections are caused by enterococci, mainly *E. faecalis* (90%) [2], [3]. The overall mortality of this disease is 11 to 35% [4]. Recommendations of the European Society of Cardiology (ESC) and the Infectious Diseases Society of America (IDSA) continue to advocate a first line therapy with amoxicillin and gentamicin for 6 weeks [4], [5]. This recommendation is poorly followed by physicians because of the cumulative toxicity of aminoglycosides and evidence from the literature that do not support the addition of an aminoglycoside to beta-lactam treatment of patients with endocarditis caused by Gram-positive cocci [6]. Following the work of Mainardi et al. [7] and Gavalda et al. [8], an alternative treatment has appeared in the recommendations cited above with the combination of amoxicillin and ceftriaxone. But clinical trials that have been conducted are uncontrolled, with a small number of patients and have not shown any improvement in mortality [9], [10]. Thus, this combination offers mostly an alternative therapy in case of contra-indication to aminoglycosides or high-level aminoglycoside resistance.

These observations emphasize the need to evaluate new therapeutic options. Daptomycin is a cyclic lipopetide and belongs to a novel class of antimicrobial agents. It has shown its efficacy *in vitro* against enterococci isolates, even those resistant to glycopeptides [11]. Animal experiments have shown that daptomycin is effective on endocarditis caused by susceptible and multidrug resistant enterococci [12], particularly with the use of higher peak concentrations obtained with the administration of 6 mg/kg once daily, compared to 3 mg/kg every 12 hours as prescribed in the late 1980s. Efficiency of daptomycin has been reported against non dividing *Staphylococcus aureus* (*S. aureus*) and high inoculum [13], [14], usually observed in sequestered infections like endocarditis [15], but data are lacking for enterococci. The effect of pH on antimicrobial activity has been reported for quinolones and aminoglycosides against *S. aureus* and *Pseudomonas aeruginosa* [16], [17]. Lamp C et al. also demonstrated in one experiment a pH dependent activity of daptomycin against *S. aureus* but high antibiotic concentrations could overcome the inhibitory effects of acidity [18].

Therefore, we planed to investigate *in vitro* activity of daptomycin, alone or in combination, against clinical strains of *Enterococcus faecalis* isolated from endocarditis. Time-kill studies with daptomycin, alone or in combination with linezolid, tigecycline, rifampicin, gentamicin, teicoplanin and amoxicillin were performed at a standard (10^6^ CFU/ml) and high (10^9^ CFU/ml) inoculum, against exponential and stationary growth-phase bacteria. We also tested nondividing *E. faecalis*. Various pH conditions were used during the assay and two concentrations for daptomycin: 60 µg/ml, that is close to the peak obtained with a 6 mg/kg dosing regimen in human, and 120 µg/ml, obtained with a higher dosage of 8 mg/kg [19]. Other antibiotics concentrations have been chosen according to their PK/PD characteristics, the recommended dosage regimen and the peak or trough concentrations obtained with it.

## Materials and Methods

### Bacterial strains

Nine strains were included in this study. Six strains (identified as the strains FG, ZM, ME, OB, FAM, LH) isolated from blood culture of patients with confirmed endocarditis according to the Dukes modified criteria [4]. One of those strains was highly resistant to aminoglycosides (strain FG). One strain was isolated from blood culture without underlying endocarditis (strain CR). Two reference strains were included in the study: JH2-2 and ATCC11700 (CNR des entérocoques, Caen, France).

### Antibiotics

Antibiotics as pure titrated powders were supplied by their respective manufacturers (amoxicillin-ceftriaxone-gentamicin by Panpharma France, linezolid and tigecycline by Pfizer France, daptomycin by Novartis France, rifampicin by Sanofi Aventis France). Various combinations were tested at fixed concentrations: daptomycin alone (60 and 120 µg/ml), amoxicillin (5 µg/ml) with ceftriaxonee (20 µg/ml), amoxicillin with gentamicin (15 µg/ml), amoxicillin with linezolid (5 µg/ml), amoxicillin with rifampicin (2,5 µg/ml), amoxicillin with teicoplanin (15 µg/ml), amoxicillin with daptomycin (60 µg/ml), daptomycin (60 and 120 µg/ml) with rifampicin (2,5 and 10 µg/ml), daptomycin (60 µg/ml) with tigecycline (2 µg/ml), daptomycin (60 µg/ml) with linezolid (5 µg/ml).

### Medium

Mueller-Hinton broth (MBH, bioMérieux, France) was used for all experiments and supplemented with calcium at a final concentration of 50 mg/l (MHBc) due to daptomycin calcium dependency for antimicrobial activity [20]. Final calcium concentrations were measured in MBHc. Nutrient restricted medium supplemented with calcium (resMEDc) was used for optical density adaptation of stationary growth phase *E. faecalis*: 1% glucose, 4% Mueller-Hinton broth and calcium 50 mg/l in phosphate buffered saline (NaCl 137 mmol/l, Na_2_HPO_4_ 10 mmol/l, KH_2_PO_4_ 2 mmol/l, pH 7,4) [10]. pH was adjusted to 5, 6 or 7 with 0,1 NaOH to a final percentage of total volume <1%.

### Susceptibility testing

The Minimal Inhibitory Concentrations (MICs) were determined by Etest (Biomérieux, France) on Mueller-Hinton Agar (calcium final concentration 50 mg/l) inoculated with a standard inoculum (10^5^ to 10^6^ CFU/ml) according to The European Committee on Antimicrobial Susceptibility Testing (EUCAST) guidelines [21]. MICs were also determined with a high inoculum (10^8^ to 10^9^ CFU/ml), incubated at 37°C for 24 hours.

### Time kill studies

Bacteria were initially cultured for 24 hours at 37°C with 10% CO_2_ on blood agar plates then subcultured on MHBc for 24 hours at 37°C and with 10% CO_2_. Those cultures were considered as stationary growth-phase bacteria and used to prepare exponential growth phase *E. feacalis* at high and standard inoculum, chemically arrested growth phase *E. faecalis* at standard inoculum and stationary growth phase *E. faecalis* at standard and high inoculum, as detailed below. Final bacteria suspensions were treated with various antibiotic combinations or daptomycin alone and cultured for 24 hours at 37°C. Culture aliquots of 100 µl were removed at 1, 4 and 24 hours and plated on agar for colony counts. A positive bactericidal activity was defined by a ≥3log_10_ reduction in colony counts. Model experiments were performed in triplicate to ensure reproducibility.

### Time kill studies with exponential growth-phase *E. faecalis*

Stationary growth-phase *E. faecalis* were prepared as detailed, resuspended in MHBc and incubated at 37°C while shaking at 250 rpm for 3 hours prior to examination. Standard inoculum of 10^6^ CFU/ml was obtained by standardizing OD_550_ to 0.125 followed by a 1∶100 dilution. High inoculum was obtained by centrifugation of bacterial suspensions (5000×g for 10 minutes) at 4°C. Centrifuged aliquots were then resuspended in MHBc for exponential testing and adjusted to an OD_550_ of 1. Antibiotics were then added to the bacterial suspension and colony counts performed as detailed.

### Time kill studies of stationary growth-phase *E. faecalis*

Initial stationary growth phase bacteria were obtained as detailed above, OD_550_ standardized to 0.125 and followed by a 1∶100 dilution to obtain a standard inoculum. Centrifugation (5000×g for 10 minutes) was performed and culture aliquots resuspended in resMHBc and adjusted to an OD_550_ of 1 to obtain high inoculum suspensions. Antibiotics were then added to the bacterial suspensions and colony counts performed as detailed.

### Time kill studies of chemically arrested growth *E. faecalis*

Exponential growth-phase *E. faecalis* were prepared as detailed and inoculum adjusted to an OD_550_ of 0.125. Carbonyl cyanide m-chlorphenylhydrazone (CCCP) was added at a final concentration of 10 µM. Bacterial suspensions were then divided into two portions, incubated 3 hours at 37°C while shaking (250 rpm).OD_550_ was adjusted to 0.125 and followed by a 1∶100 dilution for the portion used in susceptibility testing, the second being used as control to ensure cell viability.

### Statistical analysis

Changes in CFU/ml at 24 h in conditions of high inoculum were compared by Mann-Whitney *U*-test. A *p* value of ≤0.05 was considered significant. All statistical analyses were performed using GraphPad Prism software 6.0 (GraphPad, San Diego, CA, USA).

### Ethic statements

Ethical approval was waived for this study. Patient approval is not necessarily required for the use of bacteria isolated from clinical strains. French legislation exempts the work from requiring it (Law n° 2004-800 August 2004 the 6th, bioethic issues).

## Results

### Susceptibility testing

MICs were determined by Etest for amoxicillin, ceftriaxone, teicoplanin, linezolid, daptomycin and tigecycline (Table 1). All strains tested were susceptible to amoxicillin, daptomycin, teicoplanin, linezolid and resistant or intermediate for tigecycline. In conditions of high inoculum, MICs increased 2- to 8-fold for all antibiotics.

**Table 1:** Susceptibilities to antibiotics of 5 clinical strains isolated from endocarditis and 2 reference strains of *Enterococcus faecalis* in conditions of standard and high inoculum (MICs expressed in µg/ml).

Strains^c^	Amoxicillin MIC			Ceftriaxone MIC			Teicoplanin MIC			Daptomycin MIC			Tigecycline MIC			Linezolid MIC		
	Standard inoculum		High inoculum	Standard inoculum		High inoculum	Standard inoculum		High inoculum	Standard inoculum		High inoculum	Standard inoculum		High inoculum	Standard inoculum		High inoculum
JH2-2	0,32^a^	**S** b	0,75	>32	**R**	>32	1,5	**S**	3	2	**S**	2	0,75	**R**	1,5	1,5	**S**	3
ATCC 11700	0,5	**S**	1	>32	**R**	>32	0,5	**S**	3	0,38	**S**	3	1	**R**	2	1,5	**S**	4
FAM	0,38	**S**	0,5	>32	**R**	>32	0,38	**S**	3	1	**S**	4	0,38	**I**	1	1,5	**S**	4
FG	0,5	**S**	1	>32	**R**	>32	0,25	**S**	1	1,5	**S**	3	2	**R**	3	1	**S**	2
LH	0,38	**S**	1	3	**R**	>32	0,25	**S**	1,5	1,5	**S**	3	1,5	**R**	3	2	**S**	6
ME	0,25	**S**	0,38	3	**R**	>32	0,75	**S**	4	2	**S**	4	0,75	**R**	2	1,5	**S**	4
OB	0,38	**S**	0,75	>32	**R**	>32	0,19	**S**	2	0,38	**S**	3	0,38	**I**	2	0,75	**S**	3

a MICs expressed in micrograms per milliliter and determined by E-tests on Mueller-Hinton Agar, supplemented with Calcium (50 mg/l) innoculated with standard inoculum (10^5^ to 10^6^ CFU/ml) and high inoculum (10^8^ to 10^9^ CFU/ml).

b S, sensitive; R, resistant. S ans R breakpoints are determined with EUCAST 2011 breakpoints.

c 7 strains of *Enterococcus faecalis*: 5 clinical strains obtained from patients presenting endocarditis (OB, FAM, ME, LH, FG) and 2 referral strains (JH2-2, ATCC 11700).

### Exponential growth phase *E. faecalis* with a standard inoculum

Daptomycin at 60 µg/ml and the combination amoxicillin-gentamicin resulted in a 4-log decrease of the initial inoculum on all strains except the strain with high-level aminoglycosides resistance (strain FG) where the 4-log reduction was achieved in 4 to 24 hours (Table 2). There were no synergistic or antagonist effect when daptomycin was associated with other antibiotics. The combination amoxicillin-ceftriaxone was more slowly bactericidal on 6/7 strains with a 3 to 4-log reduction in 24 hours and the combination amoxicillin-teicoplanin was bactericidal at a 3-log decrease level on 4/7 strains. Both amoxicillin-linezolid and amoxicillin-rifampicin combinations were poorly bactericidal with a 1 to 2-log decrease for 5/7 and 6/7 strains respectively.

**Table 2:** Results of time kill studies with 7 strains of *Enterocossus faecalis* with a standard inoculum: 10^5–6^ CFU/ml and exponential growth phase bacteria.

Antimicrobial Agent^a^	Change in log_10_CFU/ml^b^																				
	Strains^c^																				
	OB			FAM			LH			ME			FG			ATCC 11700			JH2-2		
	1 h	4 h	24 h	1 h	4 h	24 h	1 h	4 h	24 h	1 h	4 h	24 h	1 h	4 h	24 h	1 h	4 h	24 h	1 h	4 h	24 h
A (5)+G (15)	**−4**	−4	−4	**−4**	−4	−4	**−4**	−4	−4	**−4**	−4	−4	0	0	**−4**	**−4**	−4	−4	**−4**	−4	−4
A (5)+C (20)	0	−1	**−3**	0	−1	**−3**	0	−1	**−4**	−1	−1	**−3**	−1	−1	**−4**	−1	**−4**	−4	0	0	**−4**
A (5)+R (2,5)	0	−1	−1	0	−1	−2	0	0	−2	0	−1	−2	0	0	−2	0	−1	−1	0	0	**−3**
A (5)+Te (15)	−1	−1	−2	−1	−1	−2	0	0	**−3**	−1	−1	−3	0	0	**−3**	0	−1	−2	0	0	**−3**
A (5)+L (5)	0	0	−2	0	−1	−1	0	0	−2	0	−1	−2	0	0	**−3**	0	−1	**−3**	0	0	−1
A (5)+D (60)	**−4**	−4	−4	**−4**	−4	−4	**−4**	−4	−4	**−4**	−4	−4	**−3**	−4	−4	**−4**	−4	−4	**−4**	−4	−4
D (60)	**−4**	−4	−4	**−4**	−4	−4	**−4**	−4	−4	**−4**	−4	−4	−2	**−4**	−4	**−4**	−4	−4	**−4**	−4	−4
D (60)+Ti (5)	**−4**	−4	−4	**−4**	−4	−4	**−4**	−4	−4	**−4**	−4	−4	−2	**−4**	−4	**−4**	−4	−4	**−4**	−4	−4
D (60)+R (2,5)	**−4**	−4	−4	**−4**	−4	−4	**−4**	−4	−4	**−4**	−4	−4	−2	**−4**	−4	**−4**	−4	−4	**−4**	−4	−4
D (60)+L (5)	**−4**	−4	−4	**−4**	−4	−4	**−4**	−4	−4	**−4**	−4	−4	**−3**	−4	−4	**−4**	−4	−4	**−4**	−4	−4

a A, amoxicillin; G, gentamicin; C, ceftriaxon; R, rifampicin, Te, teicoplanin; L, linezolid; D, daptomycin; Ti, tigecycline. Numbers in brackets indicate antibiotic concentration expressed in micrograms per milliliter.

b Changes from the starting inoculum expressed in log_10_CFU/ml modifications.

c 7 strains of *Enterococcus faecalis*: 5 clinical strains obtained from patients presenting endocarditis (OB, FAM, ME, LH, FG) and 2 reference strains (JH2-2, ATCC 11700).

### Chemically growth arrested bacteria under a standard inoculum

All the combinations tested, but daptomycin, showed lower kill rates when compared to exponential growth phase bacteria (Table 3). Daptomycin at 60 µg/ml and the combination amoxicillin-gentamicin resulted in a 2 to 4-log reduction in 1 hour and a 4-log reduction in 24 hours for 7/7 and 6/7 strains respectively. No synergistic or antagonist effects could be evidenced when daptomycin was associated with other antibiotics. Any other combinations tested were either bacteriostatic or ineffective on all strains, particularly the combination amoxicillin-linezolid that completely lost any activity on 3/7 strains.

**Table 3:** Results of time kill studies with 7 strains of *Enterocossus faecalis* with a standard inoculum: 10^5–6^ CFU/ml and chemically growth arrested bacteria.

Antimicrobial Agent^a^	Change in log_10_CFU/ml^b^																				
	Strains^c^																				
	OB			FAM			LH			ME			FG			ATCC 11700			JH2-2		
	1 h	4 h	24 h	1 h	4 h	24 h	1 h	4 h	24 h	1 h	4 h	24 h	1 h	4 h	24 h	1 h	4 h	24 h	1 h	4 h	24 h
A (5)+G (15)	**−3**	−4	−4	−2	**−3**	−4	**−4**	−4	−4	**−4**	−4	−4	0	0	−1	−2	**−4**	−4	−1	−2	**−4**
A (5)+C (20)	0	0	**−1**	0	0	−1	0	0	−1	0	−1	**−3**	−1	−1	−2	0	0	−1	0	0	−2
A (5)+R (2,5)	0	0	0	0	0	−1	0	0	−1	0	0	−1	0	0	−2	0	0	−1	0	0	−2
A (5)+Te (15)	0	−1	−1	0	0	−1	0	0	−2	0	−1	−2	0	0	−1	0	0	−1	0	0	−1
A (5)+L (5)	0	0	0	0	0	0	0	0	−1	0	0	−1	0	0	−1	0	0	−1	0	0	0
A (5)+D (60)	−2	**−3**	−4	−2	**−3**	−4	−2	**−4**	−4	**−4**	−4	−4	−2	−2	**−4**	−2	**−4**	−4	−2	**−3**	−4
D (60)	−2	**−3**	−4	−2	**−3**	−4	−2	**−4**	−4	**−4**	−4	−4	−2	−2	**−4**	−2	**−4**	−4	−2	**−3**	−4
D (60)+Ti (5)	−2	**−3**	−4	−1	−2	**−4**	−2	**−4**	−4	**−3**	−4	−4	−2	−2	**−4**	−2	**−4**	−4	−2	**−3**	−4
D (60)+R (2,5)	−2	−2	**−4**	−1	−2	**−4**	−2	**−3**	−4	**−3**	−4	−4	−2	−2	**−4**	−1	**−3**	−4	−2	**−3**	−4
D (60)+L (5)	−2	−2	**−4**	−1	−2	**−4**	−2	**−3**	−4	**−3**	−4	−4	−2	−2	**−4**	−1	**−3**	−4	−2	**−3**	−4

a A, amoxicillin; G, gentamicin; C, ceftriaxon; R, rifampicin, Te, teicoplanin; L, linezolid; D, daptomycin; Ti, tigecycline. Numbers in brackets give antibiotic concentration expressed in micrograms per milliliter.

b Change from the starting inoculum expressed in log_10_CFU/ml modifications.

c 7 strains of *Enterococcus faecalis*: 5 clinical strains obtained from patients presenting endocarditis (OB, FAM, ME, LH, FG) and 2 referral strains (JH2-2, ATCC 11700).

### Exponential growth phase *E. faecalis* under a high inoculum

**Figure 1:**
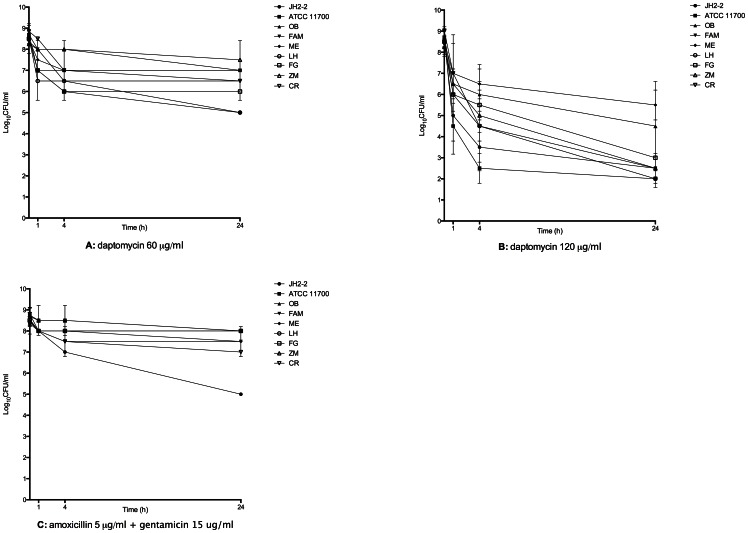
Time kill studies with 9 strains of *Enterococcus faecalis* with a high inoculum and exponential growth phase bacteria. Nine strains of *Enterococcus faecalis* were used, including: 6 clinical strains obtained from patients presenting endocarditis (OB, FAM, ME, LH, FG, ZM); 1 clinical strain obtained from enterococcus bacteriemia without endocarditis (CR) and 2 reference strains (JH2-2, ATCC 11700). Daptomycin alone at 120 µg/ml (B) was bactericidal on 8/9 strains in 1 to 4 h with a 3 to 5-log decrease and showed only a 2 log decrease after 24 hours on 1/9 strains; whereas daptomycin alone at 60 µg/ml (A) and the combination amoxicillin-gentamicin were mainly not bactericidal (C).

**Figure 2:**
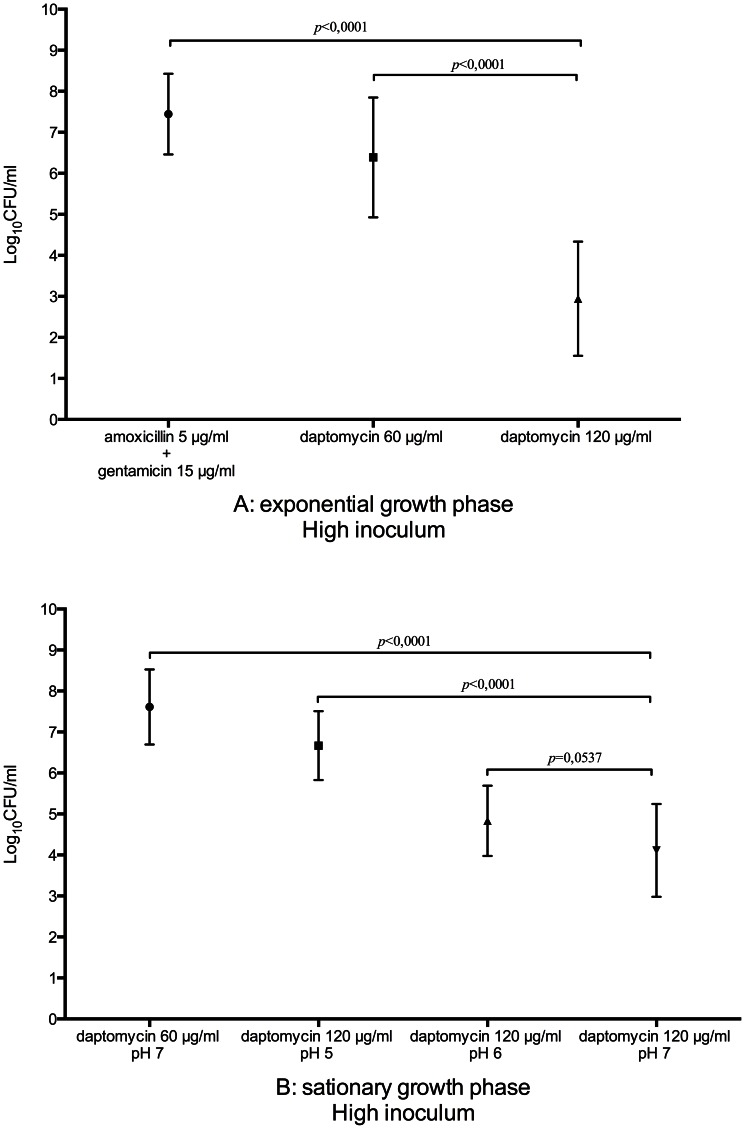
Daptomycin activity against nine strains of Enterococcus faecalis with a high inoculum and exponential or stationary growth phase bacteria, statistical analysis. Nine strains of *Enterococcus faecalis* were used, including: 6 clinical strains obtained from patients presenting endocarditis (OB, FAM, ME, LH, FG, ZM); 1 clinical strain obtained from enterococcus bacteriemia without endocarditis (CR) and 2 reference strains (JH2-2, ATCC 11700). In conditions of high inoculum and with exponential growth phase bacteria daptomycin activity at 120 µg/ml was significantly higher than daptomycin at 60 µg/ml (*p*<0,0001) and than the combination amoxicillin-gentamicin (*p*<0,0001) (A). In conditions of high inoculum and with stationary growth phase bacteria daptomycin activity at 120 µg/ml and pH 7 was significantly higher than daptomycin at 120 µg/ml and pH 5 (*p*<0,0001), than daptomycin at 60 µg/ml and pH 7 (*p*<0,0001), but not significantly higher than daptomycin at 120 µg/ml and pH 6 (*p* = 0,0537). Median changes in CFU/ml at 24 h were compared by Mann-Whitney *U*-test. A *p* value of ≤0.05 was considered significant. All statistical analyses were performed using GraphPad Prism software 6.0 (GraphPad, San Diego, CA, USA).

Daptomycin alone at 60 µg/ml showed only a bacteriostatic activity on 6/7 strains (Figure 1). Bactericidal activity was observed with 1 strain (4-log decrease in 24 hours). Daptomycin alone at 120 µg/ml was bactericidal on 8/9 strains in 1 to 4 h with a 3 to 5-log decrease and showed only a 2-log decrease after 24 hours on 1/9 strains. No synergistic or antagonist effect could be evidenced with any combination tested with daptomycin. The combination amoxicillin-gentamicin resulted in a 4-log decrease on 1/9 strains, a 2-log decrease on 2/9 strains and was totally ineffective on the 6 remaining strains. All the other combinations tested, i.e. amoxicillin-ceftriaxone, amoxicillin-teicoplanin, amoxicillin-linezolid and amoxicillin-rifampicin did not reduce initial inoculum after 24 h. Daptomycin activity at 120 µg/ml was significantly higher than daptomycin at 60 µg/ml (*p*<0,0001) and than the combination amoxicillin-gentamicin (*p*<0,0001) (Figure 2).

### Stationary growth phase *E. faecalis* under a high inoculum

**Figure 3:**
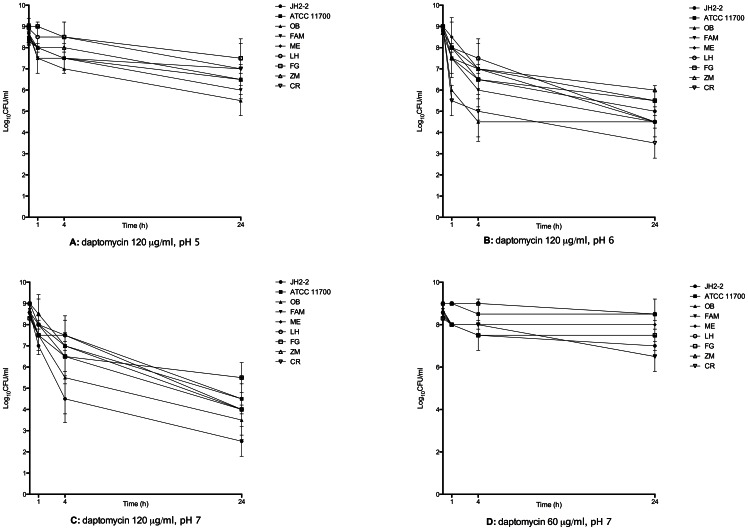
Time kill studies with 9 strains of *Enterococcus faecalis* with a high inoculum and stationary growth phase bacteria. Nine strains of *Enterococcus faecalis* were used, including: 6 clinical strains obtained from patients presenting endocarditis (OB, FAM, ME, LH, FG, ZM); 1 clinical strain obtained from enterococcus bacteriemia without endocarditis (CR) and 2 reference strains (JH2-2, ATCC 11700). Daptomycin at 120 µg/ml and pH 5 (A) was bacteriostatic on all strains (1 to 2-log reduction). When pH was adjusted to 6 (B), the activity of daptomycin at 120 µg/ml increase with a 3 to 4-log reduction in 4 hours for 3/9 strains and a ≥4-log reduction for 4/9 strains. When pH was adjusted to 7 (C), daptomycin (120 µg/ml) bactericidal activity was optimum. At 4 hours 5/9 strains experienced a 2 to 4-log decrease and at 24 hours, a 3 to 6-log reduction was evidenced for 8/9 strains. Daptomycin at 60 µg/ml and pH 7 (D), alone or in combination did not reduce initial inoculum for 4/9 strains and was bacteriostatic for 5/9 strains after 24 hours (2-log reduction).

Daptomycin at 60 µg/ml and pH 7, alone or in combination, did not reduce initial inoculum for 4/9 strains and was bacteriostatic for 5/9 strains after 24 hours (2-log reduction) (Figure 3). Daptomycin at 120 µg/ml and pH 5 was bacteriostatic on all strains (1 to 2-log reduction). When pH was adjusted to 6, the activity of daptomycin at 120 µg/ml increase with a 3 to 4-log reduction in 4 hours for 3/9 strains and a ≥4-log reduction for 4/9 strains. When pH was adjusted to 7, daptomycin (120 µg/ml) bactericidal activity was optimum. At 4 hours 5/9 strains experienced a 2 to 4-log decrease and at 24 hours, a 3 to 6-log reduction was evidenced for 8/9 strains. Daptomycin activity at 120 µg/ml and pH 7 was significantly higher than daptomycin at 120 µg/ml and pH 5 (*p*<0,0001), than daptomycin at 60 µg/ml and pH 7 (*p*<0,0001), but not significantly higher than daptomycin at 120 µg/ml and pH 6 (*p* = 0,0537) (Figure 2).

## Discussion

Endocarditis and bone infections are specific issues as bacteria are sequestered, with high density (10^8^ to 10^10^ bacteria per g of tissue) and mostly nondividing cells encased in a biofilm matrix [8], [15], [22]. Our data indicate that, in conditions of high inoculum and with stationary growth phase *E. faecalis*, daptomycin is the only antibiotic tested that remains efficient. The bactericidal activity at 120 µg/ml seems to be pH dependent. Daptomycin has been authorised by FDA with a maximum dosage of 6 mg/kg that usually leads to a peak concentration of 85 to 95 µg/ml, whereas a dosage of 8 mg/kg can lead to peak concentrations from 110 to 120 µg/ml [19], [23]. Recent data from clinical trials in healthy subjects or retrospective analysis from infected patients suggest that daptomycin could be used at doses greater than 6 mg/kg, up to 12 mg/kg, with a good safety [24], [25]. Thus, next *in vitro* studies, animal experiments or clinical trials in the field of endocarditis or bone infections should probably consider higher dosage leading to higher concentrations of daptomycin. This consideration is also supported by the emergence of daptomycin resistance in *Staphylococcus* sp. and *Enterococcus* sp. Development of daptomycin resistance in methicillin-resistant *S. aureus* has been observed following vancomycin-unresponsive *S. aureus* bacteremia or osteomyelitis [26]. Similar observations with enterococci remain rare but clinicians should be aware of this possibility [27], [28]. Our study shows increased MICs for daptomycin in conditions of high inoculum even if all the strains would remain susceptible according to current breakpoints [21]. Emergence of enterococci with higher MICs means higher mutant prevention concentration (MPC) and higher AUC_0–24_ required to meet the AUC_0–24_/MIC target, the PK/PD parameter predictive of efficacy. The use of higher dosage, that produce higher peak concentrations and AUC_0–24_, should be able to reach MPC even in condition of high inoculum, and optimize AUC_0–24_/MIC [29]. In a recently published *in vitro* pharmacokinetic/pharmacodynamic model with simulated endocardial vegetations, Ashley D. Hall et al. have shown that vancomycin-resistant *E. faecalis* developed reduced daptomycin susceptibility with daptomycin at 6, 8 and 10 mg/kg/day but not at 12 mg/kg/day. Additionally, bactericidal activity was sustained over 96 hours with daptomycin at 10 and 12 mg/kg/day regimens but not with the 6 and 8 mg/kg/day regimens [30].

Previous *in vitro* and animal studies have identified synergistic effects of daptomycin with rifampicin, ampicillin or gentamicin on enterococci and staphylococci. In vitro studies have shown 57% to 88% synergistic effect on enterococci with rifampicin by agar diffusion, chequerboard or time-kill studies [31]. Animal models of infection have focused on *S. aureus*, mainly MRSA infections. Data are promising in experimental osteomyelitis and foreign body infections but remain controversial for experimental endocarditis [22], [32], [33]. In clinical practice, rifampicin is commonly used in combination for bone and joint infections, or endocarditis on prosthetic valves where it is recommended in first line therapy for staphylococci [4]. It penetrates the biofilm matrix, and combination with daptomycin could prevent the emergence of rifampicin resistance [34], [35]. Our *in vitro* study failed to show any synergistic effects between daptomycin and all the other antibiotics tested, particularly in conditions of high inoculum and with stationary growth phase bacteria where rifampicin was used at 2,5 µg/ml and 10 µg/ml with daptomycin at 60 µg/ml and 120 µg/ml. In conditions of standard inoculum and with exponential growth phase bacteria daptomycin alone achieved a 4 log_10_ reduction when the first colony count was performed at 1 h, reaching the limit of detection with the method described. As a consequence, a potential synergistic effect with tigecycline, rifampicin and linezolid could not be evidenced with this method.

Our study has also shown a pH dependent activity of daptomycin. Lamp K. et al. have previously reported the same observation on *S. aureus* strains where increasing pH increased activity of daptomycin [18]. Daptomycin mechanism of action may help understand this phenomenon. Daptomycin inhibits formation of peptidoglycan by inhibiting transport of amino acid precursor that is pH dependent. Our results show that daptomycin, in conditions of high inoculum and with stationary growth phase bacteria, is bacteriostatic at pH 5, partially bactericidal at pH 6 and fully bactericidal at pH 7. This data is important for *in vitro* studies with daptomycin that should monitor the pH closely to determine correctly this antibiotic efficiency. *In vivo* sensitivity to daptomycin is also probably pH dependent, an interesting data for the treatment of bone infections where acidic pH is already problematic for the use of aminoglycosides. Clinical reports and literature reviews have shown the eventual efficacy of daptomycin in such infections [36], [37]. These conflicting data with those from in vitro observations show once again that our *in vitro* observations cannot be extrapolated to clinical scale. More, one has to remind that daptomycin remains bactericidal on high inoculum, stationary growth phase bacteria, and bacteria embedded in biofilm what is likely to occur in prosthetic and bone infections. Additional *in vitro* studies will be needed to better understand the mechanisms involved, such as those involved in the action of aminoglycosides on intracellular bacteria and in acidic environments [38].

Our study has limitations. We have used high concentrations of daptomycin as some data indicate that daptomycin activity is not limited to the drug free-fraction [39], [40]. Nevertheless, concentrations used in our experiments correspond to peak level concentrations but such levels are not maintained throughout the dosing interval in vivo, as they are in the in vitro time kill testing.

Therapy of enterococcal endocarditis due to amoxicillin sensitive, aminoglycosides sensitive or highly resistant strains remain controversial. Recommendations of the European Society of Cardiology (ESC) or the Infectious Diseases Society of America (IDSA) still recommend a first line therapy with amoxicillin and gentamicin for 6 weeks that is poorly followed by physicians. The efficiency of daptomycin on enterococcal infections has been proved *in vitro*, in animal infection models and our study gives positive results on high inoculum and stationary growth phase *E. faecalis*. Additional animal models should be considered now to confirm these data.

(This work was presented in part as a poster at the 51st Interscience Conference on Antimicrobial Agents and Chemotherapy, Chicago, 17 to 20 September 2011, presentation number E-1314)
